# Modulation of Inflammatory Responses by a Non-Invasive Physical Plasma Jet during Gingival Wound Healing

**DOI:** 10.3390/cells11172740

**Published:** 2022-09-02

**Authors:** Benedikt Eggers, Matthias Bernhard Stope, Jana Marciniak, Alexander Mustea, James Deschner, Marjan Nokhbehsaim, Franz-Josef Kramer

**Affiliations:** 1 Department of Oral, Maxillofacial and Plastic Surgery, University Hospital Bonn, 53111 Bonn, Germany; 2 Department of Gynecology and Gynecological Oncology, University Hospital Bonn, 53127 Bonn, Germany; 3 Department of Orthodontics, University Hospital Bonn, 53111 Bonn, Germany; 4 Department of Periodontology and Operative Dentistry, University Medical Center of the Johannes Gutenberg University, 55131 Mainz, Germany; 5 Section of Experimental Dento-Maxillo-Facial Medicine, University Hospital Bonn, 53111 Bonn, Germany

**Keywords:** non-invasive physical plasma, cold atmospheric plasma, dentistry, gingival keratinocytes, gingival fibroblasts, cytokines, viability, wound healing, inflammation, in vitro

## Abstract

Gingival wound healing plays an important role in the treatment of a variety of inflammatory diseases. In some cases, however, wound healing is delayed by various endogenous or exogenous factors. In recent years, non-invasive physical plasma (NIPP), a highly reactive gas, has become the focus of research, because of its anti-inflammatory and wound healing-promoting efficacy. So far, since NIPP application has been poorly elucidated in dentistry, the aim of this study was to further investigate the effect of NIPP on various molecules associated with inflammation and wound healing in gingival cells. Human gingival fibroblasts (HGF) and human gingival keratinocytes (HGK) were treated with NIPP at different application times. Cell viability and cell morphology were assessed using DAPI/phalloidin staining. Cyclooxygenase (COX)2; tumour necrosis factor (TNF); CC Motif Chemokine Ligand (CCL)2; and interleukin (IL)1B, IL6 and IL8 were analysed at the mRNA and protein level by a real-time PCR and ELISA. NIPP did not cause any damage to the cells. Furthermore, NIPP led to a downregulation of proinflammatory molecules. Our study shows that NIPP application does not damage the gingival tissue and that the promotion of wound healing is also due to an anti-inflammatory component.

## 1. Introduction

The gingiva is the key barrier to physical, chemical and microbial stimuli and, thus, its integrity is important for the entire organism [1]. While keratinocytes are the main cells in the multi-layered squamous epithelium, fibroblasts are the predominant cell type in the connective tissue of the gingiva. Both tissues are separated by a basal lamina [2]. The healing of the gingiva is an evolutionary benefit and helps to maintain the barrier function to prevent further damage to underlying tissues [3]. In dental surgery, only a functioning wound healing of the soft tissue is the first sign of a successful intervention, as it provides the condition for the successful healing of the underlying hard tissue.

The process of wound healing is typically represented by four subsequent but overlapping stages. First, haemostasis occurs within the first hours after the loss of tissue integrity. This is followed by the inflammatory phase, which is characterised by an influx of leucocytes within the first three days. A three-week proliferation phase follows, which serves for tissue regeneration. Finally, the remodelling phase begins, during which the extracellular matrix is restructured for up to 12 months [4,5]. The inflammatory phase is especially important to remove dead cells and detritus and to protect the opened vulnerable defect from invasion by pathogens through an inflammatory response [4]. Nevertheless, it also subsides rapidly so as not to interfere with subsequent healing phases. Different molecules such as cyclooxygenase (COX)2, but also inflammatory cytokines and chemokines such as tumour necrosis factor (TNF); CC Motif Chemokine Ligand (CCL)2; and interleukin (IL)1B, IL6 or IL8 are primarily involved in these processes. They do not only serve as immunological components, but also play an important role in wound contraction or tissue remodelling in later phases [6,7,8,9,10]. CCL2 and IL6 thus influence neovascularisation and collagen accumulation [11,12]. Additionally, IL8 plays an important role in promoting angiogenesis and keratinocyte migration [13]. Several of these cytokines have also been shown to be released by keratinocytes [14]. In particular, the cytokines IL1, IL6, and TNF secreted by epithelial cells contribute to the improved differentiation and migration of keratinocytes in the wound area [15]. Both IL1 and TNF increase the mitosis of fibroblasts and MMP production by fibroblasts and macrophages. This is important for tissue remodelling, as effective wound healing requires a fine-tuned balance of collagen production, deposition and degradation [16]. In addition, IL1 plays a critical role in stimulating fibroblasts to release growth factors, which enhance keratinocyte proliferation and motility [17,18]. Cytokines released by keratinocytes also induce antimicrobial processes during wound closure, as long as the tissue is not fully intact [19]. In pathological conditions the inflammation state remains constant, leading to impaired wound healing that results, for example, in chronic wounds or excessive scarring. This is not only a burden on the patient’s mental health, but also an economic burden on the healthcare system [20,21]. A prolonged inflammatory phase is characterised by an increased number of inflammatory cells with neutrophils and macrophages [22]. Thus, controlling microbial contamination or reducing inflammation may help to promote a normal wound healing process [20].

In recent years, the use of Non-Invasive Physical Plasma (NIPP), a highly reactive and electrically conductive gas at room temperature, has been shown to have an overall beneficial effect on gingival wound healing [23,24]. In addition to its wound-healing effects, it is also known to reduce the bacterial load in chronic wounds [25]. Several devices have been developed for medical applications: dielectric barrier devices (DBD), plasma jets and hybrid devices, generating NIPP by the ambient air or certain inert gases, such as argon or helium [26,27]. The treatment with NIPP, a short procedure of only a few minutes, is almost painless and is so far without any described side effects. In dentistry, the potential use of NIPP is particularly attractive and promising due to the shortness of clinical interventions and the local character of the application. As well as patients with wound healing disorders, almost all patients could benefit from anti-inflammatory and anti-microbial effects and, therefore, improved wound healing. For example, NIPP has been applied after subgingival instrumentation in periodontally diseased patients [28]. Furthermore, NIPP can be used for wound healing after the transplantation of palatal soft tissues (free gingival grafts) [24]. In addition, NIPP could also be used for the healing of avulsed teeth, since we have shown beneficial effects of NIPP on regeneration-associated cementoblast functions [29]. Moreover, due to the beneficial effects of NIPP on all soft and hard tissue cells [30,31,32], wound healing after oral surgical procedures such as simple tooth extractions, implantations, surgical tooth removals, apicectomies or cystectomies is a potential area of NIPP application. In a previous study, we showed that NIPP promotes wound healing in gingival cells and tissues biopsies [30]. Cytokines and chemokines are known to play a significant role in wound healing processes. The modulation of their expression by NIPP treatment may, therefore, represent a crucial mechanism of action of NIPP-induced wound healing and tissue regeneration. The aim of this in vitro follow-up study was to investigate the influence of NIPP on proinflammatory markers in gingival cells during wound healing. Our hypothesis was that NIPP is capable of downregulating the critical mediators of inflammation.

## 2. Materials and Methods

### 2.1. Cell Culture

Human gingival fibroblasts (HGF) (HGF-1; CRL-2014; ATCC (Manassas, VA, USA) were propagated in Dulbecco’s Modified Essential Medium (DMEM; Invitrogen, Waltham, MA, USA), supplemented with 10 % fetal bovine serum (FBS; Invitrogen), and 1 % antibiotics (penicillin/streptomycin; Invitrogen) using a humidified incubator (Thermo Fisher Scientific, Waltham, MA, USA) at 37 °C, 5 % CO_2_, and 95 % humidity. Human gingival keratinocytes (HGK) (primary gingival keratinocytes; PCS-200-014; ATCC) were propagated in Keratinocyte Growth Medium 2 (PromoCell, Heidelberg, Germany) using equal conditions of cultivation, containing 1 % antibiotics (Invitrogen). The culture medium was exchanged every second or third day. For the experiments, the cells were seeded into 3.5 cm petri dishes at a density of 5 × 10^4^ cells each. (VWR, Radnor, PA, USA). For equalising the growth rate of both cell types, the FBS concentration of full HGF medium was reduced to 1 % 24 h prior to the experiments.

### 2.2. NIPP Application

**Figure 1:**
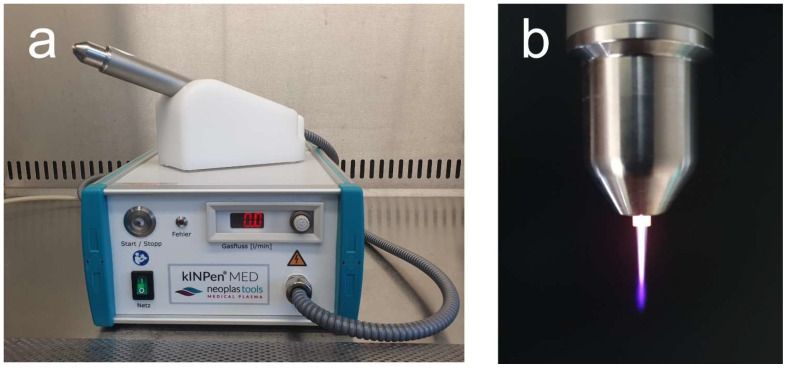
The NIPP device used for the experiment was the argon based kinpen med (neoplas med, Greifswald, Germany). (**a**) Operation unit; (**b**) handpiece with NIPP effluent.

NIPP was generated with the argon-based Kinpen Med (neoplas med, Greifswald, Germany) at 4.0 standard litres per minute (Figure 1a,b). Cells were treated for 30 s, 60 s, and 90 s, as previously described at a distance of 2 cm [30].

### 2.3. Immunofluorescence

For the fluorescence analysis, HGF and HGK cells were seeded on glass coverslips (Thermo Fisher Scientific), propagated and treated with NIPP, as described above. One d and 2 d after NIPP treatment, the cells were fixed with 4 % paraformaldehyde (Sigma-Aldrich, Saint-Louis, MO, USA) for 10 min, washed with PBS and permeabilized in 0.1 % Triton X-100 (Sigma-Aldrich) for 5 min. Subsequently, the cells were incubated for 60 min with phalloidin (Sigma-Aldrich, 100 µM) for actin filament labelling. After rinsing, the cells were stained with DAPI (Sigma-Aldrich, 1 µg/mL) for 5 min to label the DNA. Finally, the stained cells were mounted with Mowiol (Carl-Roth GmbH, Karlsruhe, Germany) and analysed using the ZOE Fluorescent Cell Imager (Bio-Rad, Hercules, CA, USA).

### 2.4. Analysis of mRNA Levels

For RNA isolation, the RNeasy Mini Kit (Qiagen, Hilden, Germany) was used according to the manufacturer’s instructions. Subsequently, 1 µg of the isolated RNA was reverse transcribed for cDNA synthesis using the iScript Select cDNA Synthesis Kit (Bio-Rad Laboratories, Munich, Germany). A mixture of 1 µL of cDNA with 12.5 µL of SsoAdvanced Universal SYBR Green Supermix (Bio-Rad); 2.5 µL of commercially available primers (GAPDH, COX2, TNF, CCL2, IL1B, IL6, IL8; QuantiTect Primer Assay, Qiagen); and 9 µL of deionised water per sample was finally amplified using the iCycler iQ5 detection system (Bio-Rad), with the protocol below: 95 °C for 5 min, followed by 40 cycles of denaturation at 95 °C for 10 s and combined annealing/extension at 60 °C for 30 s. The results were analysed by applying the comparative threshold cycle method.

### 2.5. Analysis of Protein Levels

The protein levels of COX2 were analysed in cell lysates at 1 d and 2 d with a specific COX2 ELISA Kit (Bio-Techne; Minneapolis, MN, USA) according to the manufacturer’s instructions. TNFA, CCL2, IL1B, IL6, IL8 protein levels were analysed in cell culture supernatant at 1 d and 2 d using a specific enzyme-linked immunoassay (ELISA) Kits (Bio-Techne) according to the manufacturer’s instructions. A microplate reader (Epoch™ Microplate Spectrophotometer, BioTek Instruments, Winooski, VT, USA) was used to measure the optical density at 450 nm. The results were normalised to the total protein concentration. For this purpose, the Pierce BCA Protein Assay Kit (23227, Thermo Scientific, Pierce Biotechnology, Rockford, IL, USA) was used with the absorbance measured at 570 nm, as described above.

### 2.6. Statistical Analysis

The statistical analysis was performed using GraphPad Prism version 7 software (GraphPad Software, Inc., La Jolla, CA, USA) by applying the Kruskal–Wallis test with a post hoc Dunn’s multiple comparisons test. *p*-values less than 0.05 were considered significant. All experiments were performed in triplicates and repeated at least twice.

## 3. Results

### 3.1. Impact of NIPP on Cell Viability and Cytoskeleton Morphology

**Figure 2:**
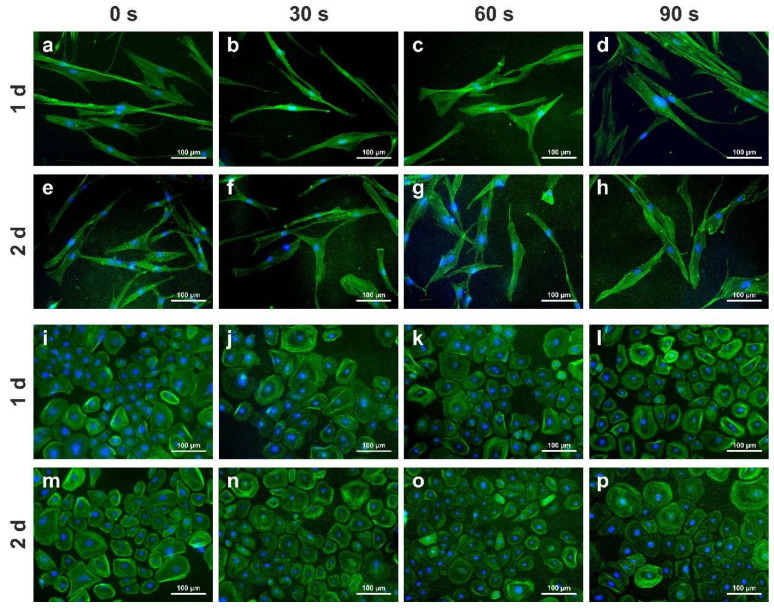
Effect of NIPP on cell viability and cytoskeleton architecture using double staining with DAPI and phalloidin. Human Gingival Fibroblasts (HGF) (**a**–**h**) and Human Gingival Keratinocytes (HGK) cells (**i**–**p**) were treated with NIPP for the indicated time intervals and analysed at 1 d and 2 d. The scale bars represent 100 μm.

First, we aimed to determine whether NIPP exerts any effects on cell viability and cytoskeleton architecture. As evidenced by phalloidin staining, the cytoskeleton of both HGF and HGK cells remained intact after NIPP applications over varying treatment durations. All the cells remained viable (Figure 2a–p).

### 3.2. Effects of NIPP on Regulation of COX2

**Figure 3:**
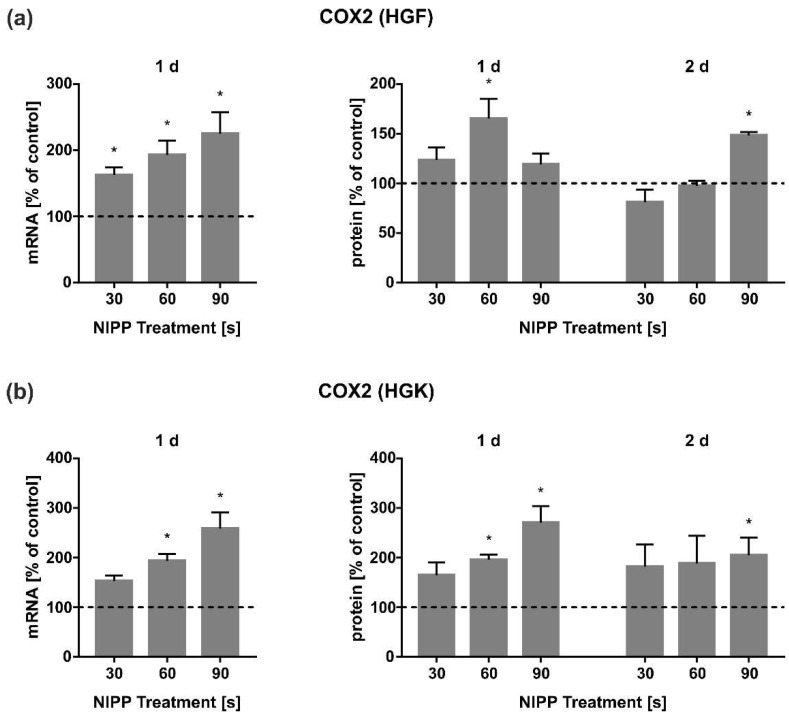
Effect of NIPP on COX2 expression in HGF and HGK cells at mRNA level after 1 d (*n* = 9) and at the protein level after 1 d and 2 d (*n* = 6). (**a**) COX2 regulation in HGF; (**b**) COX2 regulation in HGK. Controls were set to 100 % (dashed line). * Statistically significant from untreated control (*p* < 0.05).

Additionally, we investigated the influence of NIPP on the regulation of key markers in wound healing at both mRNA and protein level. First, we examined COX2 as one of the major regulators in the initial phase of inflammatory processes. NIPP led to a dose-dependent upregulation of mRNA expression in HGF and HGK cells (Figure 3a,b). An upregulation of intracellular COX2 was also observed at the protein level after 1 d and 2 d, with the highest expression levels achieved at 1 d for both cell types (Figure 3a,b).

### 3.3. Effects of NIPP on Regulation of TNF

**Figure 4:**
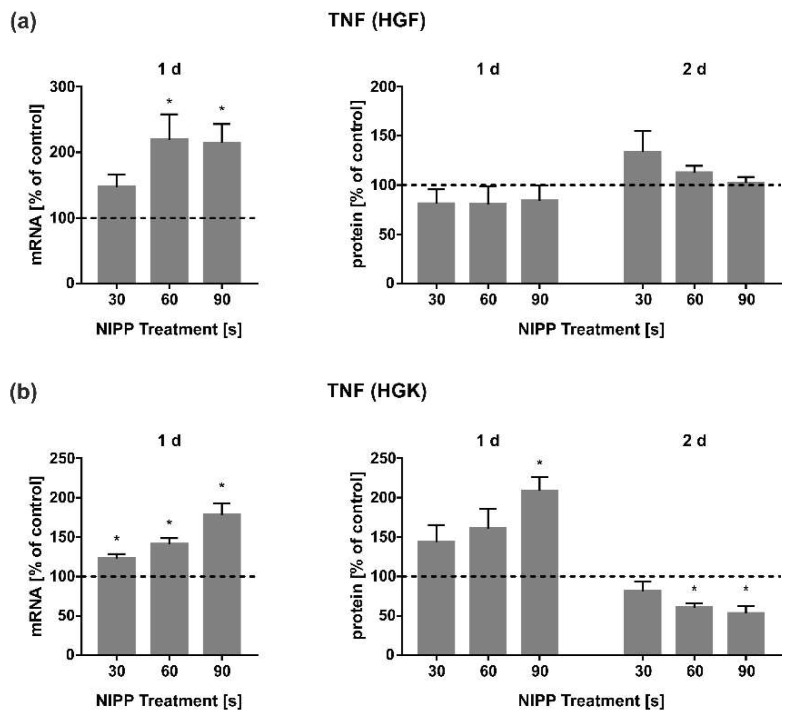
Effect of NIPP on TNF expression in HGF and HGK cells at mRNA level after 1 d (*n* = 9) and at protein level after 1 d and 2 d (*n* = 6). (**a**) TNF regulation in HGF; (**b**) TNF regulation in HGK. Controls were set to 100 % (dashed line). * Statistically significant from untreated control (*p* < 0.05).

A further central cytokine in inflammatory processes and wound healing represents TNF. The application of NIPP led to an upregulation of TNF mRNA after 1 d in HGF and HGK cells (Figure 4a,b). A clear dose dependency was observed, especially in HGK cells (Figure 4b). Interestingly, this upregulation of TNF expression in HGF cells was not detectable at the level of secreted proteins at 1 d. However, a slight upregulation was observed at 2 d after 30 s and 60 s of NIPP application (Figure 4a). Interestingly, in HGK cells, on the other hand, an upregulation of TNF at protein level was observed after 1 d and a significant downregulation after 2 d (Figure 4b).

### 3.4. Effects of NIPP on Regulation of CCL2

**Figure 5:**
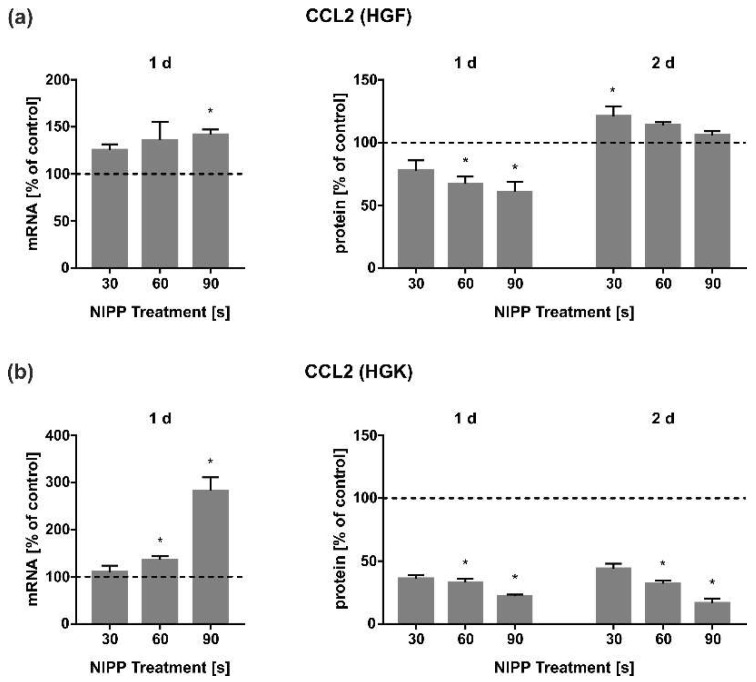
Effect of NIPP on CCL2 expression in HGF and HGK cells at the mRNA level after 1 d (*n* = 9) and at the protein level after 1 d and 2 d (*n* = 6). (**a**) CCL2 regulation in HGF; (**b**) CCL2 regulation in HGK. Controls were set to 100 % (dashed line). * Statistically significant from untreated control (*p* < 0.05).

Next, we analysed the regulation of CCL2, a very potent chemokine that, amongst other activities, supports inflammatory processes by recruiting leucocytes. CCL2 was induced by NIPP at the mRNA level in both HGF and HGK cells and reached the peak after 90 s of NIPP treatment (Figure 5a,b). Interestingly, this stimulatory effect could not be observed at the extracellular protein level, neither in HGF nor in HGK cells (Figure 5a,b). Surprisingly, NIPP caused even a significant decrease in CCL2 protein at some time points.

### 3.5. Effects of NIPP on Regulation of IL1B

**Figure 6:**
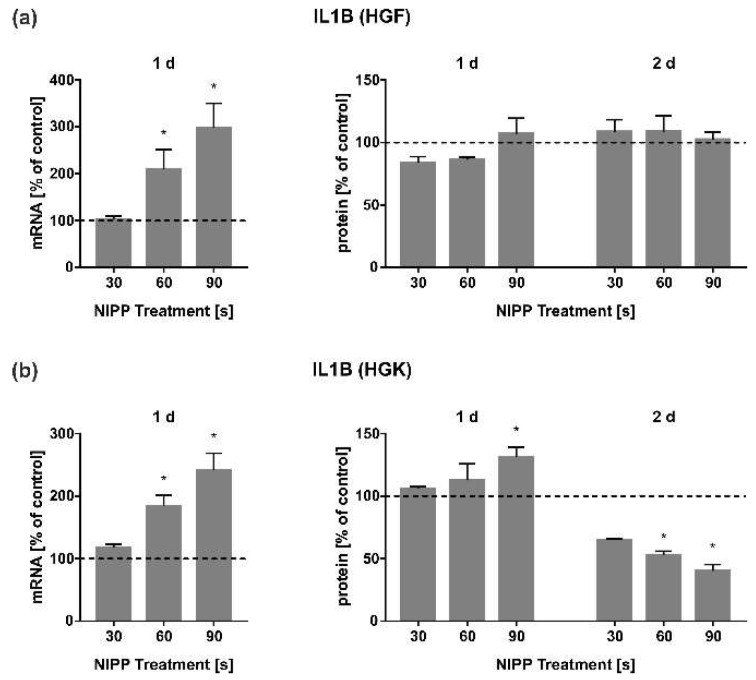
Influence of NIPP on IL1B expression in HGF and HGK cells at the mRNA level after 1 d (*n* = 9) and at the protein level after 1 d and 2 d (*n* = 6). (**a**) IL1B regulation in HGF; (**b**) IL1B regulation in HGK. Controls were set to 100 % (dashed line). * Statistically significant from untreated control (*p* < 0.05).

Following CCL2, we focused on inflammatory cytokine IL1B. NIPP treatment had a very strong dose-dependent effect on the mRNA expression of this cytokine after 1 d in both HGF and HGK cells (Figure 6a). However, this strong regulation was not reflected at the level of secreted proteins in HGF cells (Figure 6a). IL1B was even strongly downregulated in a dose-dependent manner in the HGK cells’ protein level at 2 d, as compared to untreated cells (Figure 6b).

### 3.6. Effects of NIPP on Regulation of IL6

**Figure 7:**
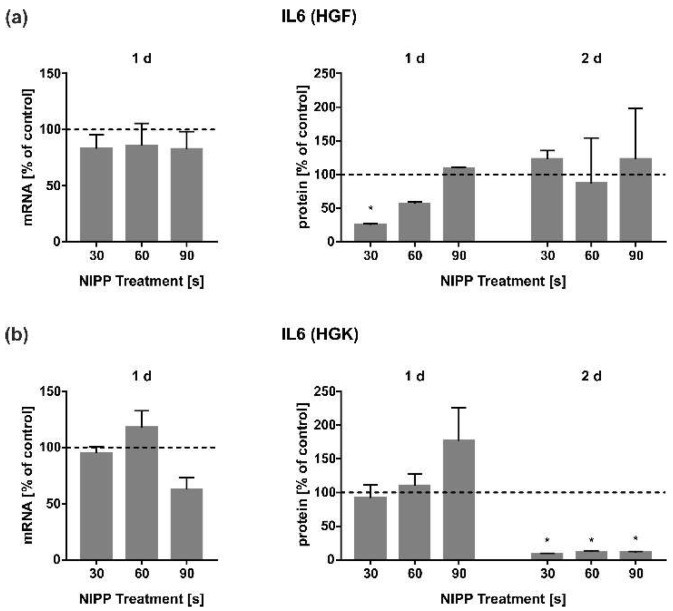
Influence of NIPP on IL6 expression in HGF and HGK cells at the mRNA level after 1 d (*n* = 9) and at the protein level after 1 d and 2 d (*n* = 6). (**a**) IL6 regulation in HGF; (**b**) IL6 regulation in HGK. Controls were set to 100 % (dashed line). * Statistically significant from untreated control (*p* < 0.05).

The regulation of IL6 was subsequently studied. The mRNA regulation of IL6 was weakly pronounced in both HGF and HGK cells (Figure 7a,b). Interestingly, at the extracellular protein levels, NIPP led to the strong downregulation of IL6 protein in HGF cells at 1 d after 30 s and 60 s NIPP-treatment and at 2 d in HGK cells. (Figure 7a,b).

### 3.7. Effects of NIPP on Regulation of IL8

**Figure 8:**
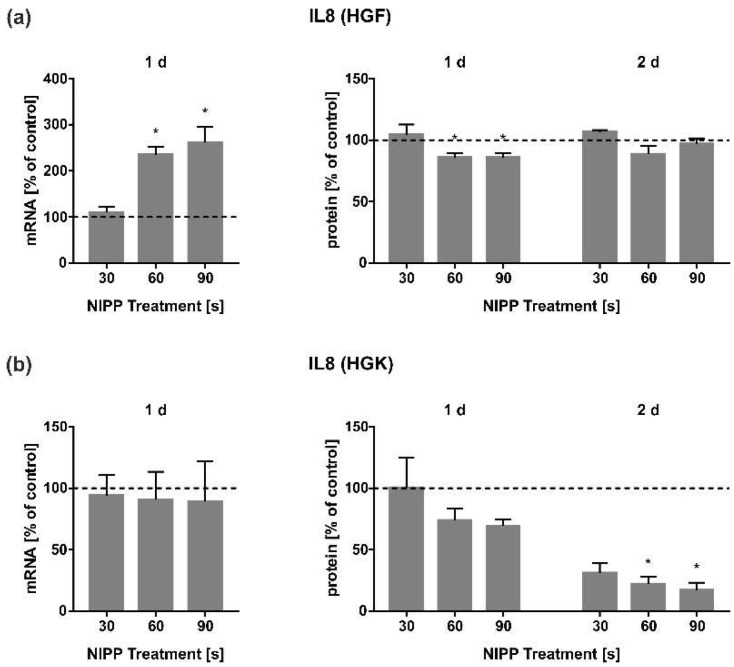
Influence of NIPP on IL8 expression in HGF and HGK cells at the mRNA level after 1 d (*n* = 9) and at the protein level after 1 d and 2 d (*n* = 6). (**a**) IL8 regulation in HGF; (**b**) IL8 regulation in HGK. Controls were set to 100 % (dashed line). * Statistically significant from untreated control (*p* < 0.05).

Finally, we analysed IL8 regulation. In contrast to IL6, in HGF cells it was shown that the IL8 mRNA was induced in a dose-dependent manner after 1 d (Figure 8a). For IL6, however, there was no regulation at the level of IL8 secretion (Figure 8a). In HGK, there was no significant regulation at the mRNA level, but IL8 secretion was very strongly downregulated in cell culture supernatants over 1 d and 2 d (Figure 8b).

## 4. Discussion

**Figure 9:**
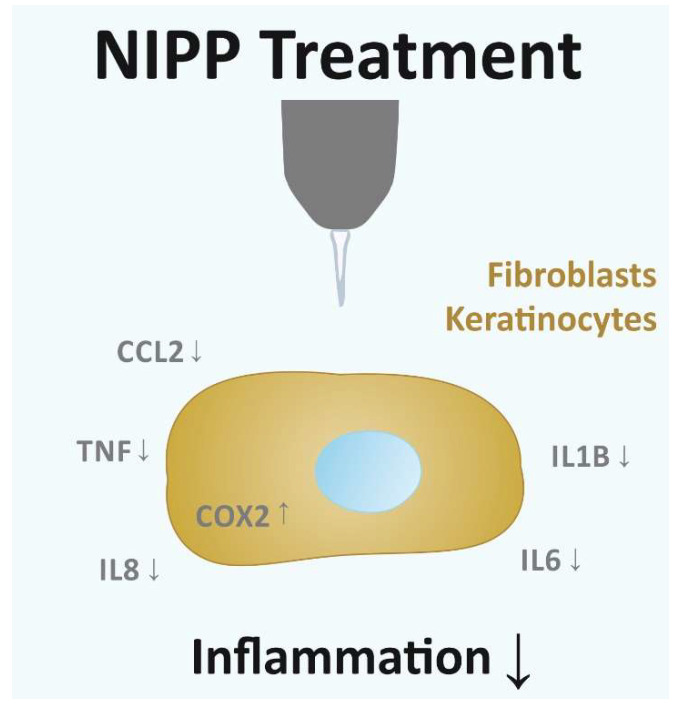
Summary: NIPP treatment of gingival fibroblasts and keratinocytes cells did not cause reduced cell viability and resulted in suppression (↓) of pro-inflammatory signal molecules and induction (↑) of regenerative COX2.

In the present study, we observed a regulatory effect of NIPP on the proinflammatory molecules involved in gingival wound healing. Although the expression of these factors was partially increased by NIPP, the application of cold plasma to HGK and HGF cells resulted in the reduced protein levels of these inflammatory mediators, suggesting an anti-inflammatory effect (Figure 9). The positive effect on wound healing that NIPP is known for, as presented in the previous studies [30,32], could, therefore, be at least partly due to the anti-inflammatory NIPP efficacy.

First, we demonstrated that NIPP has no negative effect on cell viability and cytoskeleton architecture. This confirms the results of our previous study, where it was shown that NIPP has no apoptotic effect on the cells [30]. In previous studies, however, we showed that NIPP causes changes in the cell morphology of osteoblast-like cells and murine cementoblasts with more filo- and lamellipodia, being associated with an increased migration rate [29,33]. In contrast, other authors have shown that murine C3H/10T1/2 cells on NIPP-treated surfaces exhibit less cell spreading [34]. In the present study, we did not detect a change in cell morphology. However, the contradictory results in the literature could be due to the use of different devices (DBD vs. plasma jet), different experimental set-ups, and different treatment durations and incubation times. In addition, different cell types from different tissues and species were used for the described studies.

In the present study, we focused on the regulation of COX2, which is an inducible enzyme associated to inflammatory response [35]. Additionally, COX2 is also mainly involved in the wound healing of soft and hard tissue [36,37]. In our experiments, we could observe an upregulation of COX2 mRNA. Although an increase in COX2 at the protein level was also observed after NIPP treatment, this was not significant in gingival cells in most cases. (Figure 3a,b). Our results confirm the findings of previous studies on human periodontal ligament (PDL) and osteosarcoma cells (MG63), which have also shown a stimulatory effect of NIPP on COX2 [31,32]. However, these works used a dielectric barrier discharge and not a plasma jet device such as the one applied in the present study. This allows the conclusion that we may be dealing with a general and device-independent effect of NIPP treatment on COX2 induction by NIPP. COX2 is also an essential enzyme in the conversion of arachidonic acid to prostaglandin E2 [38], which is directly related to tissue damage by altering the metabolic processes in connective tissue and promoting osteoclastic bone resorption [39,40]. Thus, it is possible that the COX2 increase could also be induced by the tissue damage per se [41,42]. However, we can exclude this in our in vitro approaches, as shown by the fluorescence analyses of NIPP treated cells (Figure 2a,b). Moreover, in our previous study, the caspase-3 activity of HGK and HGF cells remained unaltered after NIPP treatment [30]. Nevertheless, it is conceivable that molecular healing processes induced by tissue damage are additionally enhanced by NIPP treatment. This can only be clarified in subsequent ex vivo or in vivo models.

In addition, we focused on the regulation of TNF, a proinflammatory cytokine that plays a crucial role in inflammatory processes [43]. Furthermore, TNF is very important in the early phase of wound healing, modulating collagen synthesis, fibroblast proliferation and angiogenesis [44,45]. Depending on the phase of the healing process, however, increased TNF levels can improve wound healing on the one hand, but also worsen it on the other [46,47]. In our experiments, TNF expression was increased in a dose-dependent manner by NIPP-treatment in HGF and HGK cells (Figure 4a,b). Similar effects of NIPP on TNF regulation have been observed in PDL cells using a different device (DBD) [32]. Moreover, in an animal model, NIPP also regulated TNF levels during wound healing at 2 d [48]. In contrast, in osteosarcoma cells TNF was not modulated by NIPP at 1 d [49]. Interestingly, other studies also demonstrated that prostate cancer cells show no change in TNF protein levels 4 h after NIPP application [50]. Both studies used a technical identical precursor model of the plasma device that we used, but with a slightly reduced gas flow of three standard litres/minute. Different cell types and different tissues appear to respond differently to NIPP application. However, one reason could also be the treatment time, as in the studies that showed no effect of plasma on TNF regulation, NIPP was only applied for a short time (10 s; lower gas flow). Further studies are needed to clarify this. Interestingly, in the present study, NIPP had no significant effect on the regulation of TNF protein in HGF cells, even though the expression of TNF was altered in the presence of NIPP. In HGK cells, after the initial upregulation by NIPP, there were even significantly lower protein levels compared to the untreated control after 2 d (Figure 4a,b). The discrepancy between the transcriptional and protein levels of TNF could possibly be due to post-transcriptional regulation mechanisms mediated by NIPP [51]. For inflammatory factors, it has been described that RNA-binding proteins can regulate stability and the translational efficiency of the targeted mRNA [52]. Moreover, NIPP could have affected protein levels in the supernatant by the inhibition of TNF secretion. Further experiments are necessary to clarify this possibility.

In the present study, CCL2 was also regulated by NIPP treatment. NIPP resulted in a dose-dependent upregulation of CCL2 expression, whereas a slight decrease in CCL2 protein levels was observed in both HGF and HGK cells after 1 d. This reduction was even more pronounced in HGK cells after 2 d (Figure 5a,b). CCL2 is an important chemokine in early inflammation, being essential for monocyte recruitment and the activation of macrophage. Therefore, CCL2 represents a key molecule of inflammatory reactions to microbial pathogens and traumatic insults, as well as in wound healing [53,54]. CCL2 has been shown to improve wound healing by increasing the expression of growth factors [11]. The CCL2 regulation by NIPP in wound healing processes has also been described by other investigators. Arndt et al. observed an induction of CCL2 protein by NIPP in dermal fibroblasts after 1 d and 2 d [55]. Similar to TNF, we also found a rather inhibitory effect of NIPP on CCL2 protein regulation. Our opposite observation as compared to the study by Arndt et al. could be explained by the use of dermal instead of gingival fibroblasts in their study. Additionally, in their study, cells were exposed to a plasma torch in contrast to our study, where a plasma jet was applied [55]. Further studies applying plasma jet and plasma torch devices to the same cell type are needed to detect differential CCL2 regulation. The fact that CCL2 was downregulated by NIPP in our study again indicates the anti-inflammatory effect of NIPP.

Furthermore, we focused on the regulation of IL1B expression after NIPP treatment. IL1 is a cytokine that is released by many cell types and plays an important role in acute phase reactions in particular [56]. Like other cytokines, it is also important for the process of wound healing by controlling, for example, angiogenesis [57]. Moreover, IL1 is also found in high concentrations in burnt or chronic wounds, and targeting this cytokine improves wound healing [58,59]. In our study, IL1B in HGK and HGF cells was upregulated at transcriptional level but downregulated at protein level. The NIPP-induced upregulation of IL1B as observed in the present study was also found in PDL and osteosarcoma cells [31,32,49]. The downregulation of IL1B protein in the present study again underlines the anti-inflammatory effect of NIPP. A reduction in IL1B protein levels with a simultaneous improvement of wound healing by NIPP has already been shown by other authors in clinical studies and again confirms the anti-inflammatory effect of NIPP [60]. Interestingly, a 10 s application of NIPP generated by a plasma jet in prostate carcinoma cells caused no change in the protein regulation of IL1B after 4 h [50]. One reason might be, as mentioned above, the lower intensity of the gas flow, the shorter the application time or the shorter the time of incubation. Finally, however, it cannot be excluded that prostate cancer cells respond differently to NIPP than gingival cells. Further experiments with correspondingly short application and examination times on gingival cells are necessary to better understand the regulation of IL1B following NIPP treatment.

Similar effects were also shown for the other cytokines IL6 and IL8. IL6 is a proinflammatory cytokine that is released during acute-phase reactions. It is released in the early inflammation phase and contributes to the lymphocytes differentiation and leukocyte chemotaxis [61,62]. In addition, IL6 is essential for wound healing by promoting growth factor release [7,12]. Interestingly, the expression of IL6 in HGF and HGK cells was not significantly altered by NIPP. This was in contrast to observations in PDL cells in previous studies [32]. Differences could be due to the different NIPP devices used (DBD vs. plasma jet) or the different cell types. Similar to IL1B, we found an inhibitory effect of NIPP on IL6 secretion at 1 d in HGF and at 2 d in HGK cells. Similarly, other investigators have shown an anti-inflammatory effect of NIPP in periimplantitis involving the regulation of IL6 [63]. Thus, our results are in accordance with this in vivo study. Interestingly, NIPP causes an opposite regulation of IL6 in LNCaP than in PC3 cells after 4 h—both cell lines were stimulated with a plasma jet for 10 s [50]. This proves that different cells react differently to the influence of NIPP. For a better comparison, application times of 10 s should also be applied to gingival cells in further investigations.

Additionally, the chemokine IL8 was a focus of this study, since it is not only involved in the chemotaxis of neutrophiles and acute inflammation, but is also essential for proper wound healing [13,64]. In HGK cells, IL8 levels were similar to that of IL6 after NIPP treatment. Like in dermal fibroblasts and MG63 cells, we observed a dose-dependent upregulation of IL8 expression after NIPP treatment [31,65]. Like the other cytokines, the protein levels of IL8 were reduced after NIPP treatment. This was also shown in PC3 prostate carcinoma cells using a precursor model of the plasma device we used [50]. In a clinical trial, anti-inflammatory effects related to IL8 after NIPP treatment on diabetic foot ulcers were also observed [60]. Thus, our results suggest that the anti-inflammatory effects of NIPP are also mediated by IL8 regulation in gingival cells.

In the present study, discrepancies in expression and protein levels were found for CCL2, IL1, Il6 and IL8. In general, however, the clinical effects are reflected only in protein synthesis and secretion, but not in mRNA transcription. One reason for the discrepancies between mRNA expression and protein levels could be that NIPP inhibits protein secretion. Additionally, post-transcriptional modification could also be a reason [51]. We may have discovered a new mechanism for NIPP that should be clarified in future studies. We can, therefore, conclude a strong downregulation of inflammatory markers mediated by NIPP. This anti-inflammatory effect should be further investigated in additional in vitro and ex vivo studies.

The present study was Intended as a follow-up study to the data already described in Eggers et al. 2022, in which we observed a stimulating effect of NIPP on proliferation and remodelling [30]. The proliferation-promoting effect of NIPP has also been described by other authors in in vitro [66], in vivo [67] and clinical studies [68], but the underlying mechanisms are still largely unknown. Our study demonstrates that anti-inflammatory effects are induced by NIPP even in the absence of an inflammatory state, with a simultaneous proliferation-promoting effect of NIPP. This suggests that NIPP promotes regenerative rather than reparative wound healing. Regeneration is characterized by proliferative processes in the course of which damaged cells are replaced and the tissue section concerned is functionally restored as well. This contrasts with reparative wound healing processes. These also include proliferation, but are accompanied by fibrosis, scarring, and usually a permanent change in tissue structure and functionality [69]. This is a characteristic of embryonic wounds, which are distinguished by an inflammatory reaction with considerably fewer differentiated inflammatory cells, and which, therefore, heal without scarring—in contrast to the wound healing of adult skin [70,71]. As the profile of growth factors released—for example, the significantly lower TGF-B levels in embryonic wounds—are also very different from that in adult wounds [71], this should be the focus of further investigation to better understand NIPP-mediated gingival wound healing.

Furthermore, other wound healing interventions that control cytokine release have also been described. For example, ozone, which has been demonstrated to promote wound healing, has also been shown to reduce inflammatory cytokines during the healing process [72]. Additionally, laser treatment has been shown to improve wound healing by directly modulating the regulation of cytokines [73]. Comparisons between the effects of laser, NIPP, or ozone are needed to better predict the clinical value of these treatment approaches. It has to be considered that a comparison of studies with different plasma devices is only possible to a very limited extent. The technologies used for plasma generation, but also the device parameters—for example, carrier gas, flow rate, frequency—of similar devices influence the properties of the plasma and, subsequently, the ROS composition. Thus, the biological effects also vary [74]. Moreover, a frequent oversight is that malignant cells are often compared with non-malignant cells in experimental approaches. Here, the different physiological conditions can also lead to—possibly NIPP-independent—variations in cell responses. Only the NIPP treatment duration allows an approximate comparability of experiments. Finally, clinical studies with patients are the gold standard for robust statements on NIPP efficacy in the various diseases and defects. In this study, we focused on the direct application of NIPP as a simple, inexpensive and non-harmful method for improving wound healing. As we have already demonstrated a wound-healing and mineralisation-promoting effect for hard tissue in previous studies [31,75], NIPP could be used by dentists for different procedures. For example, the antimicrobial effect would allow an application in the field of immediate implantation. For successful osseointegration, the elimination of microbial infection in post-extraction sockets is important for one-step implantation. The direct application of NIPP to post-extraction sockets could minimise complications by its antimicrobial effects [76]. In addition, NIPP could accelerate the healing of the peri-implant tissues. Future in vivo studies should clarify if NIPP can be used for the treatment of these post-extraction sockets. In addition, the application in the field of periodontology would also be possible and has already been investigated in some studies in vitro and in clinical trials [28,32]. In comparison to non-chirurgical periodontal treatment, a reduced recolonisation of putative periodontopathogens has been observed [28]. Further studies, especially clinical studies, are needed to determine the value of NIPP compared to conventional therapy. Indirect applications of NIPP have also been described in the literature, which substantially expand the treatment spectrum and, for example, allow rinsing [77,78]. Thus, the effect of NIPP-treated liquids on oral cells could also be a focus of future studies.

The presented study has some limitations. It is important to keep in mind that we used commercially available cells in our study. Patient-specific primary HGF cells and primary HGK cells may show different gene expression profiles. Further studies should, therefore, investigate the inflammatory factors in primary cells in more detail. Furthermore, it must be emphasized that the analyses of the NIPP effect on gingival cells were studied only after 1 d and 2 d. Extended incubation times after NIPP treatment might allow better conclusions to be drawn. However, we focused on studying these short time points because our previous studies show that they are the most appropriate for studying the NIPP effect on hard and soft tissue cells [29,30,31,32], and this is in line with other authors [55]. In addition, the presented study aims to expand the results already published on proliferation and remodelling with the same observation time points [30]. Nevertheless, the investigation of factors of inflammation, proliferation, degradation and matrix remodelling should also be investigated for further time points, such as 3 d or 7 d, and should, thus, be a focus of further investigations. We were also only able to examine a small selection of wound healing-associated factors. Other cytokines (IL2, IL4), growth factors (EGF, KGF), and MMPs (MMP2, MMP7, MMP9) relevant to the wound healing process may also be affected by NIPP treatment. Furthermore, our hypothesis of regenerative rather than reparative wound healing mechanisms should be confirmed by further in vitro studies. Markers that are characteristic of embryonic wounds, such as TGF-B1, TGF-B2, TGF-B3 and PDGF [71], should be investigated in more detail after NIPP treatment to clarify this hypothesis. Moreover, it must be noted that only one NIPP device was used in this study. Therefore, statements on the general NIPP efficacy are only possible to a limited extent. With regard to the implementation of NIPP therapies in dentistry, other NIPP devices would also have to be characterised and compared with each other. It is possible that different devices/technologies are differently suited for different clinical applications. Finally, the effects of NIPP should be confirmed in a clinical trial to better understand the proliferation-promoting and anti-inflammatory effect of NIPP, as well as to establish the application of this non-invasive technology in dentistry.

## 5. Conclusions

Overall, our data show that NIPP treatment leads to a reduction in the protein levels of numerous inflammatory mediators in gingival cells. This anti-inflammatory effect of NIPP may be at least partly responsible for the promotion of gingival wound healing by NIPP (Figure 9). NIPP could, therefore, be a promising treatment strategy for the therapy of infected and slow-healing oral wounds.

## Data Availability

Not applicable.

## References

[1] Dutzan N., Konkel J.E., Greenwell-Wild T., Moutsopoulos N.M. (2016). Characterization of the Human Immune Cell Network at the Gingival Barrier. Mucosal Immunol..

[2] Winning T.A., Townsend G.C. (2000). Oral Mucosal Embryology and Histology. Clin. Dermatol..

[3] Landén N.X., Li D., Ståhle M. (2016). Transition from Inflammation to Proliferation: A Critical Step during Wound Healing. Cell. Mol. Life Sci..

[4] Reinke J.M., Sorg H. (2012). Wound Repair and Regeneration. Eur. Surg. Res..

[5] Gonzalez A.C.D.O., Costa T.F., Andrade Z.D.A., Medrado A.R.A.P. (2016). Wound Healing-A Literature Review. An. Bras. Derm..

[6] Hübner G., Brauchle M., Smola H., Madlener M., Fässler R., Werner S. (1996). Differential Regulation of Pro-Inflammatory Cytokines during Wound Healing in Normal and Glucocorticoid-Treated Mice. Cytokine.

[7] McFarland-Mancini M.M., Funk H.M., Paluch A.M., Zhou M., Giridhar P.V., Mercer C.A., Kozma S.C., Drew A.F. (2010). Differences in Wound Healing in Mice with Deficiency of IL-6 versus IL-6 Receptor. J. Immunol..

[8] Takamiya M., Fujita S., Saigusa K., Aoki Y. (2008). Simultaneous Detection of Eight Cytokines in Human Dermal Wounds with a Multiplex Bead-Based Immunoassay for Wound Age Estimation. Int. J. Leg. Med..

[9] Low Q.E.H., Drugea I.A., Duffner L.A., Quinn D.G., Cook D.N., Rollins B.J., Kovacs E.J., DiPietro L.A. (2001). Wound Healing in MIP-1α−/− and MCP-1−/− Mice. Am. J. Pathol..

[10] .Futagami A., Ishizaki M., Fukuda Y., Kawana S., Yamanaka N. (2002). Wound Healing Involves Induction of Cyclooxygenase-2 Expression in Rat Skin. Lab. Investig..

[11] Ishida Y., Kuninaka Y., Nosaka M., Furuta M., Kimura A., Taruya A., Yamamoto H., Shimada E., Akiyama M., Mukaida N. (2019). CCL2-Mediated Reversal of Impaired Skin Wound Healing in Diabetic Mice by Normalization of Neovascularization and Collagen Accumulation. J. Investig. Dermatol..

[12] Johnson B.Z., Stevenson A.W., Prêle C.M., Fear M.W., Wood F.M. (2020). The Role of IL-6 in Skin Fibrosis and Cutaneous Wound Healing. Biomedicines.

[13] Jiang W.G., Sanders A.J., Ruge F., Harding K.G. (2012). Influence of Interleukin-8 (IL-8) and IL-8 Receptors on the Migration of Human Keratinocytes, the Role of PLC-γ and Potential Clinical Implications. Exp. Med..

[14] Nowinski D., Höijer P., Engstrand T., Rubin K., Gerdin B., Ivarsson M. (2002). Keratinocytes Inhibit Expression of Connective Tissue Growth Factor in Fibroblasts in Vitro by an Interleukin-1alpha-Dependent Mechanism. J. Investig. Derm..

[15] Sivamani R.K., Garcia M.S., Isseroff R.R. (2007). Wound Re-Epithelialization: Modulating Keratinocyte Migration in Wound Healing. Front. Biosci..

[16] Mast B.A., Schultz G.S. (1996). Interactions of Cytokines, Growth Factors, and Proteases in Acute and Chronic Wounds. Wound Repair Regen..

[17] Maas-Szabowski N., Shimotoyodome A., Fusenig N.E. (1999). Keratinocyte Growth Regulation in Fibroblast Cocultures via a Double Paracrine Mechanism. J. Cell Sci..

[18] Maas-Szabowski N., Stark H.J., Fusenig N.E. (2000). Keratinocyte Growth Regulation in Defined Organotypic Cultures through IL-1-Induced Keratinocyte Growth Factor Expression in Resting Fibroblasts. J. Investig. Derm..

[19] Jiang Y., Tsoi L.C., Billi A.C., Ward N.L., Harms P.W., Zeng C., Maverakis E., Kahlenberg J.M., Gudjonsson J.E. (2020). Cytokinocytes: The Diverse Contribution of Keratinocytes to Immune Responses in Skin. JCI Insight.

[20] Mustoe T.A., O’Shaughnessy K., Kloeters O. (2006). Chronic Wound Pathogenesis and Current Treatment Strategies: A Unifying Hypothesis. Plast. Reconstr. Surg..

[21] Posnett J., Franks P.J. (2008). The Burden of Chronic Wounds in the UK. Nurs. Times.

[22] Falanga V. (2005). Wound Healing and Its Impairment in the Diabetic Foot. Lancet.

[23] Jahandideh A., Amini M., Porbagher H., Amini M. (2021). Evaluating the Effect of Cold Plasma on the Healing of Gingival Wound. J. Diabetes. Metab. Disord..

[24] Pekbağrıyanık T., Dadas F.K., Enhoş Ş. (2021). Effects of Non-Thermal Atmospheric Pressure Plasma on Palatal Wound Healing of Free Gingival Grafts: A Randomized Controlled Clinical Trial. Clin. Oral Investig..

[25] Isbary G., Heinlin J., Shimizu T., Zimmermann J.L.L., Morfill G., Schmidt H.-U., Monetti R., Steffes B., Bunk W., Li Y. (2012). Successful and Safe Use of 2 Min Cold Atmospheric Argon Plasma in Chronic Wounds: Results of a Randomized Controlled Trial. Br. J. Dermatol..

[26] Haralambiev L., Wien L., Gelbrich N., Lange J., Bakir S., Kramer A., Burchardt M., Ekkernkamp A., Gümbel D., Stope M.B. (2020). Cold Atmospheric Plasma Inhibits the Growth of Osteosarcoma Cells by Inducing Apoptosis, Independent of the Device Used. Oncol. Lett..

[27] Shimatani A., Toyoda H., Orita K., Hirakawa Y., Aoki K., Oh J.-S., Shirafuji T., Nakamura H. (2021). In Vivo Study on the Healing of Bone Defect Treated with Non-Thermal Atmospheric Pressure Gas Discharge Plasma. PLoS ONE.

[28] Küçük D., Savran L., Ercan U.K., Yarali Z.B., Karaman O., Kantarci A., Sağlam M., Köseoğlu S. (2020). Evaluation of Efficacy of Non-Thermal Atmospheric Pressure Plasma in Treatment of Periodontitis: A Randomized Controlled Clinical Trial. Clin. Oral Investig..

[29] Eggers B., Marciniak J., Deschner J., Stope M.B., Mustea A., Kramer F.-J., Nokhbehsaim M. (2021). Cold Atmospheric Plasma Promotes Regeneration-Associated Cell Functions of Murine Cementoblasts In Vitro. Int. J. Mol. Sci..

[30] Eggers B., Stope M.B., Marciniak J., Götz W., Mustea A., Deschner J., Nokhbehsaim M., Kramer F.-J. (2022). Non-Invasive Physical Plasma Generated by a Medical Argon Plasma Device Induces the Expression of Regenerative Factors in Human Gingival Keratinocytes, Fibroblasts, and Tissue Biopsies. Biomedicines.

[31] Eggers B., Marciniak J., Memmert S., Kramer F.J., Deschner J., Nokhbehsaim M. (2020). The Beneficial Effect of Cold Atmospheric Plasma on Parameters of Molecules and Cell Function Involved in Wound Healing in Human Osteoblast-like Cells in Vitro. Odontology.

[32] Kleineidam B., Nokhbehsaim M., Deschner J., Wahl G. (2019). Effect of Cold Plasma on Periodontal Wound Healing-an in Vitro Study. Clin. Oral Investig..

[33] Eggers B., Marciniak J., Memmert S., Wagner G., Deschner J., Kramer F.-J., Nokhbehsaim M. (2021). Influences of Cold Atmospheric Plasma on Apoptosis Related Molecules in Osteoblast-like Cells in Vitro. Head Face Med..

[34] Eisenhauer P., Chernets N., Song Y., Dobrynin D., Pleshko N., Steinbeck M.J., Freeman T.A. (2016). Chemical Modification of Extracellular Matrix by Cold Atmospheric Plasma-Generated Reactive Species Affects Chondrogenesis and Bone Formation. J. Tissue Eng. Regen. Med..

[35] Kurumbail R.G., Stevens A.M., Gierse J.K., McDonald J.J., Stegeman R.A., Pak J.Y., Gildehaus D., Miyashiro J.M., Penning T.D., Seibert K. (1996). Structural Basis for Selective Inhibition of Cyclooxygenase-2 by Anti-Inflammatory Agents. Nature.

[36] Zhang X., Schwarz E.M., Young D.A., Puzas J.E., Rosier R.N., O’Keefe R.J. (2002). Cyclooxygenase-2 Regulates Mesenchymal Cell Differentiation into the Osteoblast Lineage and Is Critically Involved in Bone Repair. J. Clin. Investig..

[37] Fairweather M., Heit Y.I., Buie J., Rosenberg L.M., Briggs A., Orgill D.P., Bertagnolli M.M. (2015). Celecoxib Inhibits Early Cutaneous Wound Healing. J. Surg. Res..

[38] Ricciotti E., FitzGerald G.A. (2011). Prostaglandins and Inflammation. Arter. Thromb. Vasc. Biol..

[39] Hikiji H., Takato T., Shimizu T., Ishii S. (2008). The Roles of Prostanoids, Leukotrienes, and Platelet-Activating Factor in Bone Metabolism and Disease. Prog. Lipid. Res..

[40] Noguchi K., Ishikawa I. (2000). The Roles of Cyclooxygenase-2 and Prostaglandin E2 in Periodontal Disease. Periodontol.

[41] Lee J.-H., Kim K.-N. (2016). Effects of a Nonthermal Atmospheric Pressure Plasma Jet on Human Gingival Fibroblasts for Biomedical Application. Biomed. Res. Int..

[42] Gümbel D., Suchy B., Wien L., Gelbrich N., Napp M., Kramer A., Ekkernkamp A., Daeschlein G., Stope M.B. (2017). Comparison of Cold Atmospheric Plasma Devices’ Efficacy on Osteosarcoma and Fibroblastic In Vitro Cell Models. Anticancer. Res..

[43] Marino M.W., Dunn A., Grail D., Inglese M., Noguchi Y., Richards E., Jungbluth A., Wada H., Moore M., Williamson B. (1997). Characterization of Tumor Necrosis Factor-Deficient Mice. Proc. Natl. Acad. Sci. USA.

[44] Ritsu M., Kawakami K., Kanno E., Tanno H., Ishii K., Imai Y., Maruyama R., Tachi M. (2017). Critical Role of Tumor Necrosis Factor-α in the Early Process of Wound Healing in Skin. J. Dermatol. Dermatol. Surg..

[45] Witte M.B., Barbul A. (1997). General Principles of Wound Healing. Surg. Clin. N. Am..

[46] Siqueira M.F., Li J., Chehab L., Desta T., Chino T., Krothpali N., Behl Y., Alikhani M., Yang J., Braasch C. (2010). Impaired Wound Healing in Mouse Models of Diabetes Is Mediated by TNF-Alpha Dysregulation and Associated with Enhanced Activation of Forkhead Box O1 (FOXO1). Diabetologia.

[47] Wang X., Zhang S., Dong M., Li Y., Zhou Q., Yang L. (2020). The Proinflammatory Cytokines IL-1β and TNF-α Modulate Corneal Epithelial Wound Healing through P16Ink4a Suppressing STAT3 Activity. J. Cell. Physiol..

[48] de Souza L.B., Silva J.I.D.S., Bagne L., Pereira A.T., de Oliveira M.A., Lopes B.B., do Amaral M.E.C., de Aro A.A., Esquisatto M.A.M., Santos G.M.T.D. (2020). Argon Atmospheric Plasma Treatment Promotes Burn Healing by Stimulating Inflammation and Controlling the Redox State. Inflammation.

[49] Haralambiev L., Wien L., Gelbrich N., Kramer A., Mustea A., Burchardt M., Ekkernkamp A., Stope M.B., Gümbel D. (2019). Effects of Cold Atmospheric Plasma on the Expression of Chemokines, Growth Factors, TNF Superfamily Members, Interleukins, and Cytokines in Human Osteosarcoma Cells. Anticancer. Res..

[50] Bekeschus S., Ressel V., Freund E., Gelbrich N., Mustea A., Stope M.B. (2020). Gas Plasma-Treated Prostate Cancer Cells Augment Myeloid Cell Activity and Cytotoxicity. Antioxidants.

[51] Tian Q., Stepaniants S.B., Mao M., Weng L., Feetham M.C., Doyle M.J., Yi E.C., Dai H., Thorsson V., Eng J. (2004). Integrated Genomic and Proteomic Analyses of Gene Expression in Mammalian Cells. Mol. Cell. Proteom..

[52] Uchida Y., Chiba T., Kurimoto R., Asahara H. (2019). Post-Transcriptional Regulation of Inflammation by RNA-Binding Proteins via Cis-Elements of MRNAs. J. Biochem..

[53] Wood S., Jayaraman V., Huelsmann E.J., Bonish B., Burgad D., Sivaramakrishnan G., Qin S., DiPietro L.A., Zloza A., Zhang C. (2014). Pro-Inflammatory Chemokine CCL2 (MCP-1) Promotes Healing in Diabetic Wounds by Restoring the Macrophage Response. PLoS ONE.

[54] Elmanfi S., Zhou J., Sintim H.O., Könönen E., Gürsoy M., Gürsoy U.K. (2018). Regulation of Gingival Epithelial Cytokine Response by Bacterial Cyclic Dinucleotides. J. Oral Microbiol..

[55] Arndt S., Unger P., Berneburg M., Bosserhoff A.-K., Karrer S. (2018). Cold Atmospheric Plasma (CAP) Activates Angiogenesis-Related Molecules in Skin Keratinocytes, Fibroblasts and Endothelial Cells and Improves Wound Angiogenesis in an Autocrine and Paracrine Mode. J. Dermatol. Sci..

[56] March C.J., Mosley B., Larsen A., Cerretti D.P., Braedt G., Price V., Gillis S., Henney C.S., Kronheim S.R., Grabstein K. (1985). Cloning, Sequence and Expression of Two Distinct Human Interleukin-1 Complementary DNAs. Nature.

[57] Voronov E., Shouval D.S., Krelin Y., Cagnano E., Benharroch D., Iwakura Y., Dinarello C.A., Apte R.N. (2003). IL-1 Is Required for Tumor Invasiveness and Angiogenesis. Proc. Natl. Acad. Sci. USA.

[58] Al-Roujayee A.S. (2017). Naringenin Improves the Healing Process of Thermally-Induced Skin Damage in Rats. J. Int. Med. Res..

[59] Mirza R.E., Fang M.M., Ennis W.J., Koh T.J. (2013). Blocking Interleukin-1β Induces a Healing-Associated Wound Macrophage Phenotype and Improves Healing in Type 2 Diabetes. Diabetes.

[60] Amini M.R., Sheikh Hosseini M., Fatollah S., Mirpour S., Ghoranneviss M., Larijani B., Mohajeri-Tehrani M.R., Khorramizadeh M.R. (2020). Beneficial Effects of Cold Atmospheric Plasma on Inflammatory Phase of Diabetic Foot Ulcers; a Randomized Clinical Trial. J. Diabetes. Metab. Disord..

[61] Weissenbach M., Clahsen T., Weber C., Spitzer D., Wirth D., Vestweber D., Heinrich P.C., Schaper F. (2004). Interleukin-6 Is a Direct Mediator of T Cell Migration. Eur. J. Immunol..

[62] Wright H.L., Cross A.L., Edwards S.W., Moots R.J. (2014). Effects of IL-6 and IL-6 Blockade on Neutrophil Function in Vitro and in Vivo. Rheumatology.

[63] Zhou X., Wu D., Liang D., Zhang W., Shi Q., Cao Y. (2022). Evaluation of Modified Cold-Atmospheric Pressure Plasma (MCAP) for the Treatment of Peri-Implantitis in Beagles. Oral Dis..

[64] Harada A., Sekido N., Akahoshi T., Wada T., Mukaida N., Matsushima K. (1994). Essential Involvement of Interleukin-8 (IL-8) in Acute Inflammation. J. Leukoc. Biol..

[65] Bhartiya P., Masur K., Shome D., Kaushik N., Nguyen L.N., Kaushik N.K., Choi E.H. (2021). Influence of Redox Stress on Crosstalk between Fibroblasts and Keratinocytes. Biology.

[66] Arndt S., Unger P., Wacker E., Shimizu T., Heinlin J., Li Y.-F., Thomas H.M., Morfill G.E., Zimmermann J.L., Bosserhoff A.-K. (2013). Cold Atmospheric Plasma (CAP) Changes Gene Expression of Key Molecules of the Wound Healing Machinery and Improves Wound Healing In Vitro and In Vivo. PLoS ONE.

[67] Bekeschus S., Kramer A., Schmidt A. (2021). Gas Plasma-Augmented Wound Healing in Animal Models and Veterinary Medicine. Molecules.

[68] Stratmann B., Costea T.-C., Nolte C., Hiller J., Schmidt J., Reindel J., Masur K., Motz W., Timm J., Kerner W. (2020). Effect of Cold Atmospheric Plasma Therapy vs Standard Therapy Placebo on Wound Healing in Patients With Diabetic Foot Ulcers: A Randomized Clinical Trial. JAMA Netw. Open.

[69] Gurtner G.C., Werner S., Barrandon Y., Longaker M.T. (2008). Wound Repair and Regeneration. Nature.

[70] Redd M.J., Cooper L., Wood W., Stramer B., Martin P. (2004). Wound Healing and Inflammation: Embryos Reveal the Way to Perfect Repair. Philos. Trans. R. Soc. Lond. B. Biol. Sci..

[71] Ferguson M.W.J., O’Kane S. (2004). Scar-Free Healing: From Embryonic Mechanisms to Adult Therapeutic Intervention. Philos. Trans. R. Soc. Lond. B. Biol. Sci..

[72] Teplyakova O., Vinnik Y., Drobushevskaya A., Malinovskaya N., Kirichenko A., Ponedelnik D. (2022). Ozone Improved the Wound Healing in Type 2 Diabetics via Down-Regulation of IL- 8, 10 and Induction of FGFR Expression. Acta. Biomed..

[73] Sousa L.R., Cavalcanti B.N., Marques M.M. (2009). Effect of Laser Phototherapy on the Release of TNF-Alpha and MMP-1 by Endodontic Sealer-Stimulated Macrophages. Photomed. Laser. Surg..

[74] von Woedtke T., Reuter S., Masur K., Weltmann K.-D. (2013). Plasmas for Medicine. Phys. Rep..

[75] Eggers B., Wagenheim A.-M., Jung S., Kleinheinz J., Nokhbehsaim M., Kramer F.-J., Sielker S. (2022). Effect of Cold Atmospheric Plasma (CAP) on Osteogenic Differentiation Potential of Human Osteoblasts. Int. J. Mol. Sci..

[76] Crespi R., Capparé P., Crespi G., Lo Giudice G., Gastaldi G., Gherlone E. (2017). Immediate Implant Placement in Sockets with Asymptomatic Apical Periodontitis. Clin. Implant. Dent. Relat. Res..

[77] Jacoby J.M., Strakeljahn S., Nitsch A., Bekeschus S., Hinz P., Mustea A., Ekkernkamp A., Tzvetkov M.V., Haralambiev L., Stope M.B. (2020). An Innovative Therapeutic Option for the Treatment of Skeletal Sarcomas: Elimination of Osteo- and Ewing’s Sarcoma Cells Using Physical Gas Plasma. Int. J. Mol. Sci..

[78] Li Y., Pan J., Ye G., Zhang Q., Wang J., Zhang J., Fang J. (2017). In Vitro Studies of the Antimicrobial Effect of Non-Thermal Plasma-Activated Water as a Novel Mouthwash. Eur. J. Oral Sci..

